# PhaVIP: Phage VIrion Protein classification based on chaos game representation and Vision Transformer

**DOI:** 10.1093/bioinformatics/btad229

**Published:** 2023-06-30

**Authors:** Jiayu Shang, Cheng Peng, Xubo Tang, Yanni Sun

**Affiliations:** Department of Electrical Engineering, City University of Hong Kong, Hong Kong (SAR), China; Department of Electrical Engineering, City University of Hong Kong, Hong Kong (SAR), China; Department of Electrical Engineering, City University of Hong Kong, Hong Kong (SAR), China; Department of Electrical Engineering, City University of Hong Kong, Hong Kong (SAR), China

## Abstract

**Motivation:**

As viruses that mainly infect bacteria, phages are key players across a wide range of ecosystems. Analyzing phage proteins is indispensable for understanding phages’ functions and roles in microbiomes. High-throughput sequencing enables us to obtain phages in different microbiomes with low cost. However, compared to the fast accumulation of newly identified phages, phage protein classification remains difficult. In particular, a fundamental need is to annotate virion proteins, the structural proteins, such as major tail, baseplate, etc. Although there are experimental methods for virion protein identification, they are too expensive or time-consuming, leaving a large number of proteins unclassified. Thus, there is a great demand to develop a computational method for fast and accurate phage virion protein (PVP) classification.

**Results:**

In this work, we adapted the state-of-the-art image classification model, Vision Transformer, to conduct virion protein classification. By encoding protein sequences into unique images using chaos game representation, we can leverage Vision Transformer to learn both local and global features from sequence “images”. Our method, PhaVIP, has two main functions: classifying PVP and non-PVP sequences and annotating the types of PVP, such as capsid and tail. We tested PhaVIP on several datasets with increasing difficulty and benchmarked it against alternative tools. The experimental results show that PhaVIP has superior performance. After validating the performance of PhaVIP, we investigated two applications that can use the output of PhaVIP: phage taxonomy classification and phage host prediction. The results showed the benefit of using classified proteins over all proteins.

**Availability and implementation:**

The web server of PhaVIP is available via: https://phage.ee.cityu.edu.hk/phavip. The source code of PhaVIP is available via: https://github.com/KennthShang/PhaVIP.

## 1 Introduction

Bacteriophages, or phages for short, are viruses that can infect bacteria. They are the most widely distributed and abundant biological entities in the biosphere (Cobián Güemes et al. 2016), with an estimated population of more than $10^{31}$ particles (Lyon 2017). Phages play an important role in modulating microbial system dynamics by lysing bacteria and mediating the horizontal transfer of genetic material (Fernández et al. 2018). In addition, there are accumulating studies showing that phages have an important impact on multiple applications, such as the food industry (Brüssow and Desiere 2001), disease diagnostics (Wang and Yu 2004), engineering bacterial genomes (Menouni et al. 2015), and phage therapy (Azimi et al. 2019).

A fundamental step to promote phages’ applications in these fields is phage genome annotation. Phages’ proteins are highly diverse, and their current annotations are far from complete. For example, only 33% of proteins in the RefSeq phage protein database have annotations. The annotated phage proteins can be roughly divided into two groups: virion and non-virion proteins. Phage virion proteins (PVPs) are phage structural proteins that make up phage outer protein shells (Kabir et al. 2022). They were regarded as one major evidence in phage taxonomy classification by the International Committee on Taxonomy of Viruses (ICTV). During the infection, PVP binds to the host’s receptors, aiding the insertion of the phage’s genetic materials into the host cell. Identifying PVPs is a fundamental step to understanding their biological properties and mechanisms of host cell binding. Due to their ubiquity and functional importance, PVPs have been leveraged in multiple downstream applications. For example, PVPs can be used as marker genes in phage host prediction (Boeckaerts et al. 2021) and prophage identification within bacterial genomes (Roux et al. 2015). Although PVPs have become commonly used features in several phage analysis tasks, accumulating research on the non-PVPs show that we may underestimate their importance. For example, non-PVPs usually play key roles in phages’ lifecycles, including replication and packaging. Among non-PVPs, “integrase” and “excisionase” are two widely accepted marker genes for classifying the lifestyle of phages. Based on these marker genes, several phage lifestyle prediction methods were developed (Emerson et al. 2012; Hockenberry and Wilke 2021). In addition, some non-PVPs are important for the binding of phage tail fibers to host receptor proteins. For example, the endoglycosidase of *Salmonella virus P22* will hydrolyze lipopolysaccharide and destroy the O-specific chain for phage attachments (Steinbacher et al. 1996). What is more, understanding the non-PVPs can help utilize phages for engineering bacterial genomes (Menouni et al. 2015), regulating gene expression, and introducing novel functions to change cell physiology (Howard-Varona et al. 2017).

Because PVPs and non-PVPs have different functions, distinguishing them can extend our knowledge about phage properties and functions. Although there are experimental methods for PVP annotation, such as protein arrays and mass spectrometry, they are usually time-consuming, labor-intensive, and costly. Thus, they cannot catch up with the speed of newly identified phages by high-throughput sequencing. For example, as reported in Sinha et al. (2020), only 11% of proteins can be annotated using the mass spectrometry method. Thus, computational PVP classification is still the major choice for handling large-scale input data. The major challenge for computational PVP classification is the high diversity of proteins in phages. For example, most structural proteins encoded by tailed phages, except for portal proteins, cannot be identified through pair-wise sequence alignments. According to the latest RefSeq database downloaded before December 2022, 66% of proteins are marked as “hypothetical protein”, meaning that these proteins cannot be aligned to annotated proteins. Thus, fast and accurate computational methods to predict and classify diverged PVPs are urgently needed.

### 1.1 Related work

To overcome the challenge of high sequence diversity, machine-learning models are commonly used for classifying PVP and non-PVP. Most of these tools have been discussed and evaluated by several comprehensive reviews in the past 3 years (Meng et al. 2020; Nami et al. 2021; Kabir et al. 2022). Table 1 summarizes these tools together with their employed feature encoding and machine-learning algorithms.

**Table 1.** Summary of the existing PVP classification tools.

Group	Tools	Encoding method	Model
Traditional machine-learning	PVPred-SCM (Charoenkwan et al. 2020a)	*K*-mer frequency	SCM
	Pred-BVP-Unb (Arif et al. 2020)	Physicochemical properties	SVM
	(Ru et al. 2019)	Skip-gram	RF
	(Tan et al. 2018)	*K*-mer frequency (g-gap)	SVM
	PhagePred (Pan et al. 2018)	*K*-mer frequency (g-gap)	NB
	PVP-SVM (Manavalan et al. 2018)	*K*-mer frequency, physicochemical properties	SVM
	PVPred (Ding et al. 2014)	*K*-mer frequency (g-gap)	SVM
	(Feng et al. 2013)	*K*-mer frequency	NB
Ensemble method	iPVP-MCV (Han et al. 2021)	Position-specific scoring matrix	SVM-based ensemble model
	Meta-iPVP (Charoenkwan et al. 2020b)	Probabilistic martix	SVM, RF, NB, ANN
	(Zhang et al. 2015)	Position-specific scoring matrix and physicochemical properties	RF-based ensemble model
Deep learning	DeePVP (Fang et al. 2022)	One-hot	CNN
	VirionFinder (Fang and Zhou 2021)	One-hot and physicochemical properties	CNN
	PhANNs (Cantu et al. 2020)	*K*-mer frequency	ANN
	iVIREONS (Seguritan et al. 2012)	Single amino acid	ANN

As indicated in Table 1, four learning models (SVM, NB, RF, and SCM) are commonly used in traditional machine learning-based methods. Ensemble-based methods utilize multiple models or training sets. For example, Meta-iPVP (Charoenkwan et al. 2020b) utilizes a novel feature-representing scheme and four machine-learning algorithms to encode seven input features into a probabilistic matrix. Then, the generated probabilistic matrix is fed into the SVM model to classify PVPs. More recently, deep learning-based methods, such as VirionFinder (Fang and Zhou 2021) and DeePVP (Fang et al. 2022), have been proposed for structural protein identification. Both of them use convolutional neural networks (CNNs) as classifiers. The comparison between the existing tools showed that CNN is an effective method for extracting abstract features from biological sequences (Kabir et al. 2022).

Although these tools have achieved promising performance, they still have a couple of limitations. First, except for PhANNs (Cantu et al. 2020) and DeePVP (Fang et al. 2022), all these tools are binary identifiers, which can only classify the input proteins as PVP or non-PVP. However, a more detailed multi-class classification of PVPs is also in demand to assign proteins to well-defined annotations (i.e. major tail, minor tail, and baseplate). But the best *F*1-score of PhANNs and DeePVP on multi-class classification can only reach nearly 0.7 on the benchmark dataset. Second, the reference protein sequences employed by different tools are mostly outdated. Only PhANNs and DeePVP provided scripts for re-training or re-constructing the models as reported in Kabir et al. (2022). Lacking this function hinders many tools from achieving more generalized and robust predictions for newly discovered phages. Third, although one-hot encoding and *k*-mer frequency encoding are widely used in the PVP classification task, they both have disadvantages. For example, as indicated in Ren et al. (2022), using one-hot encoding for protein sequences will return sparse matrices, leading to the curse of dimensionality problem in the machine-learning model. *k*-mer frequency encoding fails to maintain the original amino acids’ organization in the raw sequences.

### 1.2 Overview

**Figure 1.**
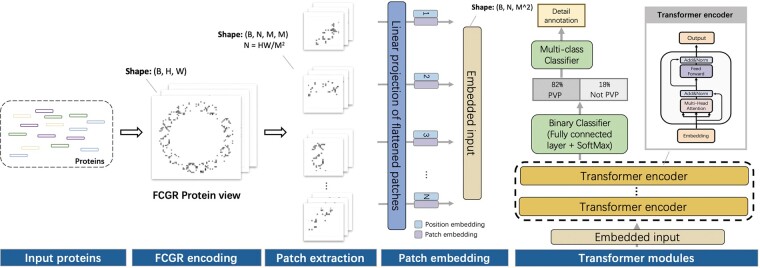
The pipelines of PhaVIP, which consists of three major stages: FCGR protein encoding, patch embedding, and Transformer modules. When taking a test/query protein as input, PhaVIP first classifies it into PVP and non-PVP. Only the predicted PVP will be classified into more detailed annotations. Dimension *B* represents the “batch size” of the input data, which means B proteins will be processed in parallel. Dimension *H* and *W* represent the height and weight of the FCGR images. Dimension *M* represents the size of the patches.

In this work, we present a method named Phage VIrion Protein (PhaVIP) for phage protein annotation. It has two functions. First, it can classify a protein into either PVPs or non-PVPs (binary classification task). Second, it can assign a more detailed annotation for predicted PVPs, such as major capsid, major tail, and portal (multi-class classification task with seven types of PVPs). To construct a complete and comprehensive dataset, we downloaded the latest annotations of phage proteins from the RefSeq database (December 2022) to train and test PhaVIP. The pipeline of PhaVIP is shown in Fig. 1. First, to address the shortages of the existing encoding methods, we employ chaos game representation (CGR) to encode proteins into images. Previous works show that using *k*-mer frequency helps distinguish proteins of different functions. However, existing models, such as CNN, are not optimized for learning the associations of *k*-mers and their frequencies. In our design, CGR can encode *k*-mer frequency into images, allowing us to leverage an image classification model, Vision Transformer (ViT) (Dosovitskiy et al. 2020; Raghu et al. 2021), from computer vision to capture and learn the patterns from CGR images. We leverage the self-attention mechanism in ViT to learn the importance of different sub-images and their associations for protein classification. In addition, because the length of the proteins varies from $10^{2}$ to $10^{3}$, applying CGR allows encoding sequences of highly different lengths into images with the same resolution. Thus, we expect that this combination can lead to better results than existing deep learning models because of the success of the ViT in image classification. In the experiments, we tested PhaVIP on multiple datasets with increasing difficulty. The comprehensive comparison with the existing methods shows that PhaVIP renders better and more robust performance. In addition, we designed two case studies to show the application of PVPs and non-PVPs for downstream phage analysis. These case studies reveal that PhaVIP can provide useful features to improve the accuracy of phage taxonomy classification and host prediction.

## 2 Materials and methods

To use machine-learning methods for classifying PVP and non-PVP, the input proteins need to be encoded into numerical values. Thus, a practical and informative sequence encoding method is crucial for classification. In this work, we applied CGR to encode protein sequences. CGR is a generalized Markov chain and allows one-to-one mapping between the image and the sequence (Löchel and Heider 2021). In addition, CGR has already shown promising results in encoding biological sequences, such as generating evolutionary trees (Hoang et al. 2016) and finding antimicrobial resistant gene (Ren et al. 2022).

Because CGR can represent protein sequences using unique images, inspired by pattern recognition problems in computer vision (CV), we apply the ViT model to extract and learn features from the CGR image. The attention mechanism in ViT can reveal the representative regions in the image and learn the associations between different parts of the image (Ghiasi et al. 2022). Several large-scale benchmark datasets in CV have shown that ViT outperforms traditional models, such as CNN, on image classification. All these features prompt us to employ ViT for PVP classification.

In the following sections, we will first introduce how CGR encodes protein sequences into unique images. Then, we will describe the ViT model optimized for the PVP classification task. Finally, we will introduce how we collect and generate the PVP datasets used in the experiments.

### 2.1 CGR encoding

**Figure 2.**
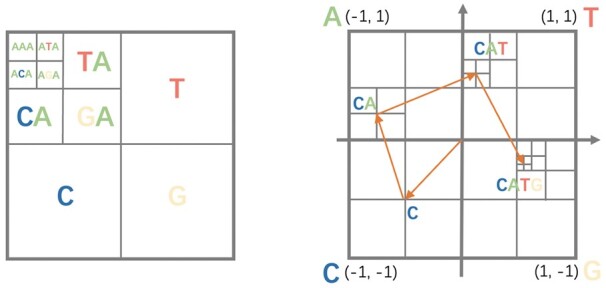
Applying CGR to a toy sequence: CATG. Left: division of the CGR space in the iterative process [reproduced from Jeffrey (1990)]; right: the process of determining the four pixels for CATG using CGR.

The CGR was first developed to construct fractals from random inputs and later extended to encode DNA sequences (Jeffrey 1990). The inputs to the CGR are sequences, and the outputs are numerical matrices/images representing the sequences. The basic idea of CGR is to map each nucleotide or amino acid to a unique coordinate in a 2D space. A toy example is given in the right panel of Fig. 2.

To encode the protein sequences into CGR images, we apply the *n*-flakes method (Fiser et al. 1994) and use frequency chaos game representation (FCGR) to produce images of the same resolution. The equations of the *n*-flakes method are given in Equation (1) (Fiser et al. 1994). *j* is the vertices ranging from 0 to *n*, which is set to 20 for amino acids. Then, FCGR can be generated by counting the points of the CGR based on a pre-defined grid. Specifically, the algorithm will split the CGR image into N×N regions. Then, the number of points in one region will be used as the region’s frequency to compress the CGR, leading to an FCGR matrix of dimension N×N for all input sequences of different lengths. The authors of Löchel and Heider (2021) show that the value in the pre-defined grid can represent the frequency of *k*-mers. For example, the gird on the top-left corner of Fig. 2 (left) represents a 3-mer “AAA”. In this work, we employ the R package “Kaos” (Löchel et al. 2020) to encode protein sequences into FCGR images. Then, we set N=64 to generate $R^{64\times 64}$ images as the representation of the protein sequences.


(1) $$\{\begin{array}{cc} V_{j}^{x}= & \;\text{sin}(\frac{2\pi j}{n})\\ V_{j}^{y}= & \;\text{cos}(\frac{2\pi j}{n}) \end{array}.$$

**Figure 3.**
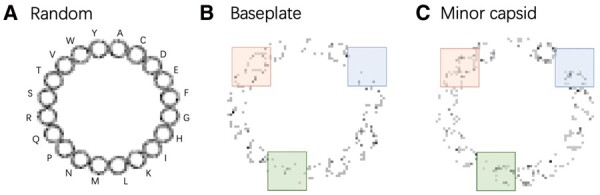
FCGR images for three sequences. (A) A random sequence. The order of vertices/amino acids is shown around the ring. (B) Baseplate protein with RefSeq accession: *YP_009788086.1*. (C) Minor capsid protein with RefSeq accession: *YP_009900655.1*. The green boxes and blue boxes in (B) and (C) show different patterns and the red boxes show exhibit patterns.

Figure 3 shows FCGR images of two different phage proteins and a random amino acid sequence. The random sequence in Panel (A) is generated by randomly choosing an amino acid for 1000 times using a uniform distribution. In contrast to the random sequence, the FCGR of baseplate protein and minor capsid protein (Fig. 3B and C) reveal more unique patterns. For example, the red patches in Fig. 3B and C exhibit a similar pattern while the blue and red patches show highly different patterns, which may signal key sequence features that can distinguish the baseplate and minor capsid proteins. The patches indicate the distribution of short motifs ending with different amino acids. These patches and their relationships/associations with other patches can be learned by our ViT model to improve classification accuracy.

### 2.2 Basic structure of ViT

After encoding the protein sequences into $R^{64\times 64}$ images, we employ ViT for PVP classification. As shown in Fig. 1, the inputs to our ViT model are FCGR images, and the output of the ViT is the probability of the protein being PVP. If the protein is predicted as PVP, our ViT will assign a more detailed annotation for the PVP.

#### 2.2.1 Patch splitting and embedding

To feed an FCGR image to ViT, we will reshape the FCGR image $G\in R^{64\times 64}$ into a sequence of flattened 2D patches $g\in R^{N\times M^{2}}$, where the dimension of each image patch is $R^{M\times M}$, and *N* is $64^{2}/M^{2}$. In our design, *M* is set to 16 by default, and the length of the input sequence *N* will be 16. Then, we can use Equation (2) to generate inputs to the Transformer model.


(2) $$\{\begin{array}{c} E_{t}=\left[g_{m}^{1}H_{e};g_{m}^{2}H_{e};\ldots ;g_{m}^{N}H_{e}\right]\\ E_{m}=IH_{m}\\ Z^{\left(0\right)}=E_{m}+E_{t} \end{array}.$$

Here, $g_{m}^{i}\in R^{1\times M^{2}}$ is the flattened 2D patch at position *i*, corresponding to a “word” token in Transformer for natural language modeling (Vaswani et al. 2017). *I* is the index of the position of each patch $x_{m}^{i}$ in the input FCGR image. $H_{e}$ and $H_{m}$ are learnable linear projection matrices for image patch and positional embedding, respectively.

#### 2.2.2 The transformer model

The architecture of the Transformer model in Fig. 1 is the same as the original design in Vaswani et al. (2017). The equations of the Transformer are listed in Equation (3). The first function is the multi-head attention mechanism (MSA layer), which can extract the importance of patches and learn their associations. Then linear projections (MLP layer) are employed to capture information from each patch simultaneously. Layer normalization (Wang et al. 2019), and residual connections (Baevski and Auli 2018) are applied before and after each block to prevent gradient exploding and gradient vanishing, respectively. In the last layer, we use the SoftMax function to estimate the probability of a protein being a PVP. If the protein is predicted as a PVP, $Z^{\left(2\right)}$ [Equation (3)] will be fed to a multi-class classifier to predict a more detailed annotation.


(3) $$\{\begin{array}{ccc} Z^{\left(1\right)} & = & \mathrm{MSA}(Z^{\left(0\right)})+Z^{\left(0\right)}\\ Z^{\left(2\right)} & = & \mathrm{MLP}(LN(Z^{\left(1\right)}))+Z^{\left(1\right)}\\ y^{\mathrm{binary}} & = & \mathrm{SoftMax}(LN(Z^{\left(2\right)}))\\ y^{\mathrm{multi}-\mathrm{class}} & = & \mathrm{SoftMax}(LN(Z^{\left(2\right)})) \end{array}.$$

#### 2.2.3 Model training

Because there are two tasks in PhaVIP: classifying the PVP and non-PVP sequences (binary classification task) and classifying seven types of PVP (multi-class classification task), we train classifiers for them separately. As introduced in Devlin et al. (2018), pre-training the Transformer model can improve the performance of the downstream task. Thus, we first apply an end-to-end method to train the binary classification model. Then, we fix the parameters in the Transformer encoder and fine-tune a new classifier layer for the multi-class classification model. Binary cross-entropy loss and L2 loss are employed for the binary classification and multi-class classification, respectively. We employ Adam optimizer with a learning rate of 0.001 to update the parameters for both tasks. The models are trained on HPC with the GTX 3080 GPU unit to reduce the running time.

### 2.3 Data collection and experimental setup

Although several PVP datasets have been constructed (Kabir et al. 2022), the latest dataset constructed by Cantu et al. (2020) was based on the protein annotations released before June 2020. In addition, some annotations of phage protein can be updated regularly in the RefSeq database. For example, as the author of DeePVP (Fang et al. 2022) reported, the protein *YP_006383517.1* was not annotated as PVP until October 2021, and this protein was re-annotated as a tail protein in the current version. Thus, in this work, we updated the PVP classification dataset by downloading all the latest annotations from the RefSeq viral protein database (December 2022). Following the guidelines of the third-party review (Kabir et al. 2022), we first recruited proteins that belong to phages. Then, the proteins with low-confidence annotations, such as “hypothetical protein”, “similar to”, “xx-like”, “unnamed”, and “putative” were removed. We extracted structural protein sequences by searching the keywords, such as “portal”, “capsid”, “tail”, “fiber”, “tape measure”, “baseplate”, and “structural”. The non-structural proteins were searched using the enzymes’ names, such as annotations ending with “ase”. In addition, we also used other keywords, such as “transcription”, “holin”, “lysin”, and “regulator”, to construct the non-PVP set. To remove the potential redundant sequences, we employed CD-HIT (Li and Godzik 2006) to cluster sequences that have above 90% similarity and used the longest sequence to represent each cluster. Finally, our dataset contains 35 213 PVP sequences and 46 883 non-PVP sequences.

#### 2.3.1 Splitting the dataset

We split our PVP dataset with increasing difficulty when constructing the training and test set. There are two tasks for PVP classification: classifying the PVP and non-PVP sequences (binary classification) and predicting the PVP types (multi-class classification). In the binary classification task, we use all the proteins for the data partition. In the multi-class classification task, we use the protein annotated with “portal”, “major capsid”, “minor capsid”, “major tail”, “minor tail”, “baseplate”, and “tail_fiber” to construct the multi-class classification dataset. According to the definition of bacteriophage assembly (Aksyuk and Rossmann 2011), tail sheath proteins are grouped into the “major tail” or “minor tail” protein classes based on their annotation in the RefSeq database. Other the remained PVPs, such as “Head-tail joining” and “collar” proteins, contain significantly fewer samples than other classes and thus are combined and labeled as “other”.

*Splitting by time*. As mentioned in Kabir et al. (2022), splitting training and test set by time is a widely used data partition method, which mimics the application scenario of using known PVPs to discover new ones. In this dataset, proteins released before December 2020 comprise the training set, while proteins released after that comprise the test set. Finally, we have 27 704 PVP sequences and 36 778 non-PVP sequences for training, and 7509 PVP sequences and 10 103 non-PVP sequences for testing in the PVP classification task. To balance the dataset, we randomly sampled non-PVP sequences to maintain the same number of samples in the binary classification as suggested in Kabir et al. (2022). In the multi-class classification, we keep the original data distribution following Cantu et al. (2020).

*Splitting by similarity*. To test PhaVIP’s performance in classifying diverged PVP, we constructed a hard case where the test sequences share low similarity with the training proteins. Specifically, we applied the all-against-all BLASTP search to our PVP dataset and calculated the product of the pair-wise identity and alignment coverage (Identity × Coverage). The identity is the percentage of exact matches in the pair-wise alignment and the coverage is the ratio of the aligned length to the length of the query sequence. This will help to estimate the global similarity of the two sequences following the recommendation by ICTV. Then, we employed the data partition strategy proposed in Petti and Eddy (2022) to create training and test data with a specified maximum similarity between train and test. In this work, we chose 0.4, 0.5, 0.6, 0.7, 0.8, and 0.9 as the thresholds and employed stratified sampling to generate six pairs of training and test sets. Based on the design of the data partition algorithm in Petti and Eddy (2022), the generated training and test set can be different for each specified similarity cutoff. The size of the train and test data can be found in the online supplementary file.

#### 2.3.2 Metrics

As mentioned in Kabir et al. (2022), the widely used metrics for evaluating PVP classification performance are precision, recall, and *F*1-score. Their formulas are listed in Equations (4) and (6):


(4) $$\mathrm{precision}=\frac{TP}{TP+FP}.$$

(5) $$\mathrm{recall}=\frac{TP}{TP+FN}.$$

(6) $$F1-\mathrm{score}=\frac{2\times \mathrm{precision}\times \mathrm{recall}}{\mathrm{precision}+\mathrm{recall}}.$$

For binary PVP classification, true positive (TP), false negative (FN), and false positive (FP) represent the number of corrected identified PVPs, the number of PVPs misclassified into non-PVPs, and the number of falsely identified PVPs, respectively. We will also report the area under the ROC curve (AUCROC) for comparison. For the multi-class classification task, we will calculate precision, recall, and *F*1-score for each class.

## 3 Results

In the experiment, we validate our pipeline on several datasets and compare PhaVIP against the state-of-the-art methods mentioned in the third-party review (Kabir et al. 2022), including VirionFinder (Fang and Zhou 2021), PhANNs (Cantu et al. 2020), DeePVP (Fang et al. 2022), Meta-iPVP (Charoenkwan et al. 2020b), PVP-SVM (Manavalan et al. 2018), and PVPred-SCM (Charoenkwan et al. 2020a). Out of these tools, only PhANNs and DeePVP provided source codes for re-training or updating the reference database. Thus, we are able to retrain PhANNs and DeePVP for both the binary and the multi-classification tasks using the suggested hyperparameters. Other tools did not provide a re-training function. Thus, we applied them to the test data directly.

In the following sections, we will first evaluate the PVP classification performance. Then, following Fang et al. (2022), we will show a case study of classifying PVP on the *mycobacteriophage* PDRPxv genome, a newly identified phage that is a candidate therapy for pathogenic *Mycobacterium*. Finally, we investigate whether using classified PVPs and non-PVPs can benefit two important phage analysis tasks: phage taxonomy classification and host prediction.

### 3.1 Performance on the benchmark dataset split by time

**Figure 4.**
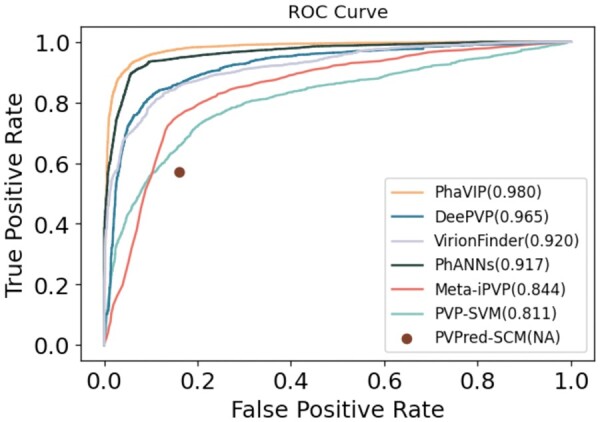
The ROC curves of the binary PVP classification on the test data by different tools. The number following the tool name is the value of the AUCROC. PVPred-SCM does not output a score for the prediction, and thus only TPR and FPR are reported.

To improve the robustness of the model, we trained PhaVIP, PhANNs, and DeePVP using 10-fold cross-validation. First, we split our training set into 10 subsets. Then, we iteratively selected nine subsets for training and one subset for validation. The 10-fold cross-validation performance of PhaVIP is shown in the online supplementary file (Supplementary Fig. S1). The model that achieves the best performance on the validation set was kept for future experiments. For other methods, we used the provided models with the suggested parameters on the test proteins. The ROC curves of all the methods on the test dataset split by time are shown in Fig. 4. The AUCROC reveals that PhaVIP has more reliable results on the dataset split by time. Because PVPred-SCM does not output a score of the prediction, we only report its recall and FP rate.

**Figure 5.**
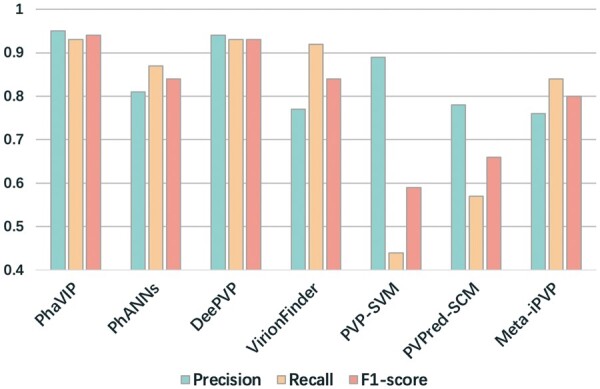
The classification performance of the binary PVP classification under the default/suggested thresholds.

In order to show the classification performance in real application scenarios, we also recorded the precision, recall, and *F*1-score of all tested tools under their default score cutoffs in Fig. 5 and Supplementary Table S1. The results reveal that PhaVIP and DeePVP have better performance than other methods on this dataset.

**Figure 6.**
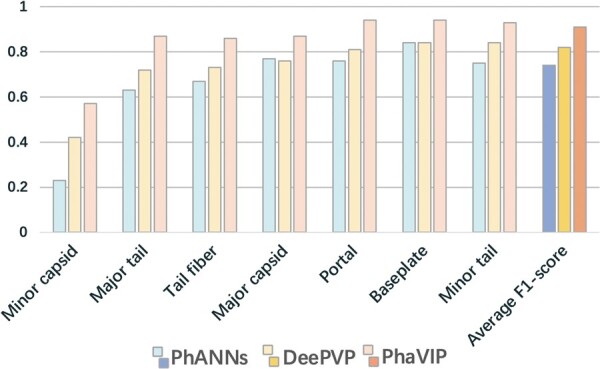
The performance of the multi-class classification. *X*-axis: the name of each PVP class. The order of the names is ranked by the class size. *Y*-axis: *F*1-score.

Next, we examine the performance of PhaVIP in the multi-class classification task. Of the available tools, only PhANNs and DeePVP enable more detailed annotation of PVPs. However, classes/labels in the original design of PhANNs and DeePVP are different from ours. Thus, we retrained both methods and compared them with PhaVIP. The *F*1-score of each class is shown in Fig. 6, and the detailed confusion matrix can be found in Supplementary Table S2. The results clearly show that the multi-class classification task is harder than classifying the PVP and non-PVP. The possible reasons are the smaller training sets and highly unbalanced classes. Although all the methods used the weighted loss method to balance the training classes, the performance of the small class (minor capsid) is still unsatisfactory. Nevertheless, PhaVIP can achieve better performance in all the classes, especially in the small ones.

### 3.2 Performance on the low-similarity dataset

**Figure 7.**
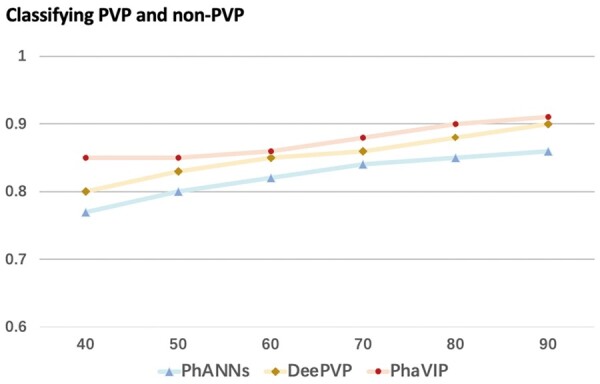
The binary classification performance on the low-similarity dataset. *X*-axis: the maximum value of identity×coverage between train and test sets. *Y*-axis: *F*1-score.

**Figure 8.**
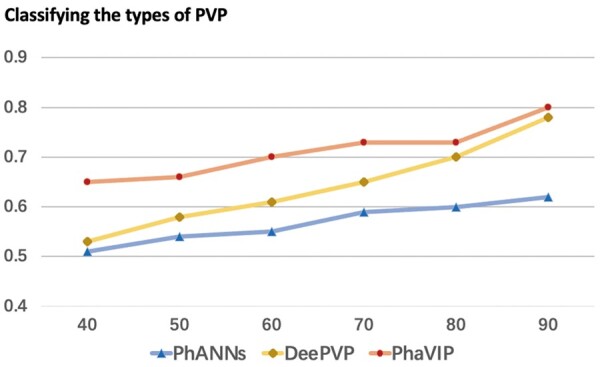
The multi-class classification performance on the low-similarity dataset. *X*-axis: the maximum value of identity×coverage. *Y*-axis: *F*1-score.

It is usually much harder to annotate diverged proteins. As mentioned in Section 2.3, we use the Identity×Coverage as the similarity measurement and control the maximum similarity between the training and test set. We generated six pairs of datasets with decreasing similarity for the binary classification task and multi-class classification task separately. The *F*1-scores of PhaVIP, PhANNs, and DeePVP are shown in Figs 7 and 8. The detailed confusion matrix of the classification can be found in Supplementary Tables S3–S14.

As expected, with the increase of the train-versus-test similarity, the *F*1-score of both methods increases. The gap between PhaVIP and the other two methods clearly reveals that our model competes favorably against the state-of-the-art methods on a wide range of similarities in both binary and multi-class classification tasks.

### 3.3 Case study: annotating proteins on the *mycobacteriophage* PDRPxv genome

Inspired by Sinha et al. (2020) and Fang et al. (2022), in this case study, we employed PhaVIP to annotate the proteins translated from *mycobacteriophage* PDRPxv, which is recently identified as a candidate therapy for *Mycobacterium*. According to Sinha et al. (2020), totally there are 107 predicted proteins in the PDRPxv genome. The authors identified 12 PVPs using the mass spectrometry method and 12 non-PVPs using the alignment method (BLAST). The functions of the other 83 proteins remain unknown. We downloaded the genomes from GenBank with accession KR029087. Because PDRPxv is not part of the RefSeq dataset, we can evaluate PhaVIP by comparing PhaVIP’s predictions with the 24 annotations derived by the mass spectrometry method and BLAST.

**Figure 9.**
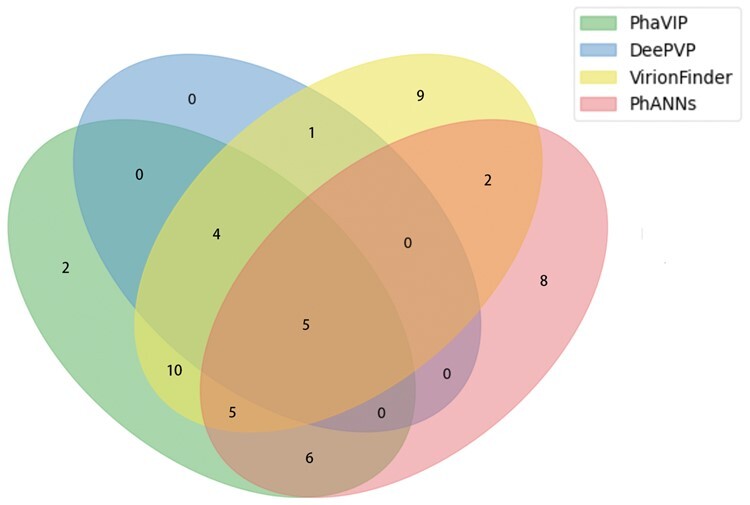
The Venn diagram of the complete PVP classification results of four best machine-leanring methods on *mycobacteriophage* PDRPxv.

We used the 24 annotated proteins as input and tested the performance of the best four tools (in the benchmark experiment in Fig. 5). As shown in Table 2, PhaVIP has better performance than other tools. In addition, all the machine learning-based methods are able to predict the function of the remaining 83 proteins, demonstrating the utility of the learning-based method for PVP classification. We used the Venn diagram to visualize the relationship between the predicted PVP sets. As shown in Fig. 9, PhaVIP, VirionFinder, and PhANNs identified more PVPs than DeePVP. This is consistent to the observation of DeePVP’s low recall in Fig. 5. In addition, 93% of PVPs predicted by PhaVIP are also classified as PVPs by other methods, which is higher than PhANNs and VirionFinder.

**Table 2.** *F*1-score of classifying proteins in *mycobacteriophage* PDRPxv genome.

Tools	PhaVIP	DeePVP	PhANNs	VirionFinder
*F*1-score	**0.88**	0.85	0.83	0.64

The highest score is highlighted in bold.

### 3.4 Using classified proteins in two important applications

It is widely known that phage proteins play essential roles in taxonomy classification and host prediction. In this section, we investigate the roles of PVPs and non-PVPs in these two tasks.

#### 3.4.1 Phage taxonomy classification

Recently, many new phages have been identified using high-throughput sequencing, especially metagenomic sequencing. vConTACT 2.0 (Eloe-Fadrosh 2019) is a widely used and robust tool for phage taxonomy classification, as reported in the phage taxonomy review (Zhu et al. 2022). It applies protein organization conservation for phage classification. Specifically, vConTACT 2.0 calculates the *P*-value that estimates the significance of two phage sequences sharing an observed number of proteins. Then, a protein-sharing network is constructed based on the *P*-value, and a clustering algorithm is applied to group “similar” sequences into the same cluster. Then, the known labels of the reference genomes in the cluster will be passed to other sequences in the same cluster.

**Figure 10.**
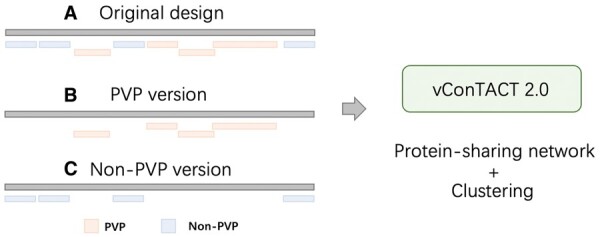
Three versions of vConTACT 2.0. (A) The original design of vConTACT 2.0 uses all the proteins from the phage genome to construct the protein-sharing network. (B) PVP version of vConTACT 2.0. (C) Non-PVP version of vConTACT 2.0.

Although vConTACT has high accuracy in classifying complete or near complete phage sequences, its running time complexity is high because of large-scale pair-wise alignments. Thus, instead of using all proteins (Fig. 10A), we propose to only use PVPs or non-PVPs to evaluate the similarity between phages. In particular, because PVPs have successful applications in phylogenetic tree construction, we expect that using just PVPs can achieve comparable accuracy of phage classification as using all proteins. Thus, in this experiment, we use just PVP or non-PVP when running vConTACT 2.0 and evaluate how PVP or non-PVP affects the classification results. First, we downloaded the benchmark dataset provided by Zhu et al. (2022). This dataset was constructed using 1460 RefSeq phage sequences from the latest ICTV 2022 taxonomy. It was split by time: 80% of the sequences in each family were used as the training set, and the remaining sequences were used as the test set. Second, we applied prodigal (Hyatt et al. 2010) to predict and translate proteins from the phage genomes in training and test sets. PhaVIP is then employed to annotate each protein. Finally, we used predicted PVPs and non-PVPs to predict the taxonomy via vConTACT, respectively. Figure 10B and C sketched the pipelines.

**Figure 11.**
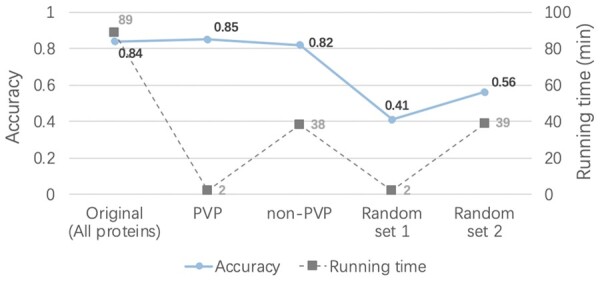
vConTACT taxonomy classification results using different sets of proteins. “Random set 1” and “Random set 2” represent randomly selected protein sets, which have the same number of proteins as PVP and non-PVP set, respectively.

The taxonomy classification results in Fig. 11 show that the PVP version of vConTACT 2.0, which only used PVP for taxonomy classification, can achieve almost the same performance as the regular vConTACT 2.0. In addition, because PVP only accounts for nearly 1/5 of the total predicted proteins, using PVP for taxonomy classification can reduce the running time significantly. Because running PhaVIP only takes about 7 min for all proteins, even with the preprocessing by PhaVIP, the total running time of taxonomy classification by vConTACT 2.0 reduces from 89 to 9 min. Using non-PVP for taxonomy classification can also reduce the running time. But the accuracy is 3% lower than using PVP.

A fair question is whether using any set of randomly chosen proteins can achieve similar accuracy with reduced running time. To answer this question, we randomly chose the same number of proteins as the PVP set and non-PVP set for taxonomy classification, respectively. In this experiment, PVP and “Random set 1” contain 7105 proteins, and non-PVP and “Random set 2” contain 29 321 proteins. The results in Fig. 11 indicate that using a random set of proteins cannot achieve comparable accuracy as using just PVPs. In addition, vConTACT 2.0’s results using “Random set 1” is worse than “Random set 2” probably because the number of proteins in “random set 2” is larger than “Random set 1”. Overall, these results show that PhaVIP can help select a small subset of important proteins for taxonomy classification.

#### 3.4.2 Phage host prediction

The hosts of the phages are mainly bacteria. Identifying the phage–host relationship helps decipher the dynamic relationship between microbes. In addition, because of the fast rise of antibiotic-resistant pathogens, phage therapy has become a potential alternative to antibiotics for killing the “superbugs” (Lee et al. 2019). Thus, predicting the phage host is important to both fundamental research and phages’ applications.

**Figure 12.**
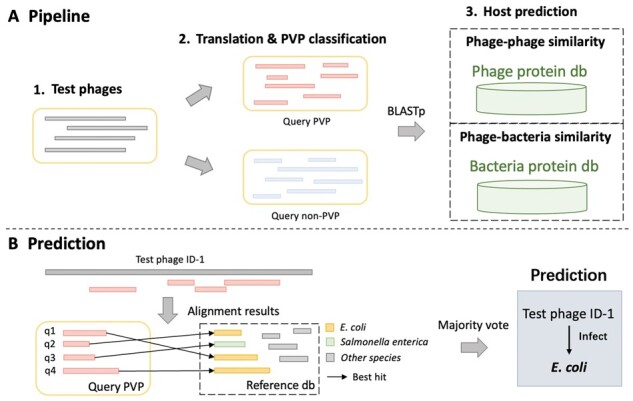
The pipeline of using similarity search for host prediction. (A) The similarity search based host prediction. We implemented two pipelines using phage protein and bacterial protein as the reference databases, respectively. (B) The majority vote method for generating the final host prediction.

As reported in Shang and Sun (2022), sequence similarity can be utilized for host prediction. If two phages share similar protein organizations, they tend to infect the same host. In addition, sequence similarity between phages and bacteria may help host prediction because phages can mobilize host genes (Howard-Varona et al. 2017). Thus, we developed a host prediction pipeline based on protein similarity in order to investigate how different types of proteins affect the prediction performance. The sketch of the pipeline is shown in Fig. 12.

First, we downloaded the widely used benchmark dataset for host prediction (Shang and Sun 2021, 2022). The training set contains 1306 phage–host interactions collected in and before 2015, and the test set contains 634 interaction pairs after 2015. Every phage is unique, and some of them infect the same host. The training and test sets share 59 host species. Because the alignment-based method cannot predict new labels, we only keep 423 phages in the test set that infect these 59 species for this experiment.

**Figure 13.**
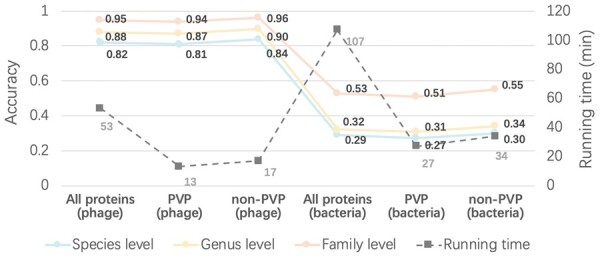
Host prediction results after PVP classification. “(phage)” and “(bacteria)” refer to the similarity search against the phage protein and bacterial protein databases, respectively.

Second, we create the reference protein databases using the predicted proteins from all the phages in the training set and their hosts. As shown in Fig. 12A, we save the proteins from phages and their hosts in two databases, respectively. Each protein has a taxonomic label. A phage protein’s label is determined by its host. A bacterial protein’s label is from itself. When there is a query/test phage, we predict its proteins and annotate PVP and non-PVP using PhaVIP. Then, we align the PVP proteins to the phage and bacterial protein databases and record each PVP’s best alignments against two databases, respectively. The labels of the best aligned proteins are used for host prediction. Because there are multiple proteins, we applied the majority vote as shown in Fig. 12B. Specifically, the label with the most votes is assigned as the host of the phage. An example is given in Fig. 12B, where three proteins were labeled as *Escherichia coli* and one protein was labeled as *Salmonella enterica*. Thus, the final predicted host of this phage is *E.coli*. Because we have two different databases, we record the results using the phage database and bacterial database separately. As a control experiment, we also repeated the host prediction process using only non-PVPs and all proteins. The host prediction results at different ranks from species to family are shown in Fig. 13.

The results reveal that the similarity search against the phage protein database always has better performance than against the bacterial protein database. This phenomenon is also noted by the existing host prediction tools. As reported in Shang and Sun (2022), the tools based on phage–phage similarity usually have better performance than those based on phage–bacteria similarity in the experiments. In addition, we found that non-PVP can achieve better performance in host prediction tasks across different taxonomy levels and databases. A plausible explanation is that the host cell attachment process is complicated and involves many proteins. Some non-PVPs, such as endoglycosidase and endosialidase (Steinbacher et al. 1996; Stummeyer et al. 2005), also play key roles in the infection, and they are likely to be host-specific. Therefore, using just PVP for host prediction does not necessarily produce a better result, which does not agree with some previous conclusions (Boeckaerts et al. 2021). We may have underestimated the importance of non-PVPs in host prediction tasks.

## 4 Discussion

In this work, we present a novel PVP classification tool, named PhaVIP, that combines CGR and ViT for protein encoding and PVP classification. PhaVIP has two functions: predicting the PVP and non-PVP and predicting the type of the PVP. CGR-based encoding can convert proteins with different lengths into images with the same resolution. For each protein, it embeds the *k*-mer frequency into a unique image, allowing us to employ the state-of-the-art image classification model, ViT, to learn the importance and associations between different parts of a CGR image. As shown in all of our experiments, ViT shows better and more robust performance in both binary classification and multi-class classification tasks. We also demonstrated that phage taxonomy classification and host prediction can benefit from using classified proteins rather than all proteins.

Although PhaVIP has greatly improved PVP classification, there are still some limitations. First, because most existing works do not provide source codes, we can only retrain the two latest published tools (DeePVP and PhANNs) in the experiments. Second, there are still other types of virion proteins, such as head–tail joining and collar proteins. However, because the number of these proteins is far fewer than in other classes, adding these classes for training may aggravate the data imbalance problem. According to our experiments, PhaVIP, DeePVP, and PhANNs cannot perform well in these small classes. For example, PhaVIP, DeePVP, and PhANNs achieved 54%, 50%, and 27% *F*1-score on the collar proteins. Thus, we have several goals to optimize or extend PhaVIP in our future work. First, although PhaVIP can perform well in binary classification, there is still room to improve multi-class classification, especially in the low-similarity data. We will investigate whether some multi-objective loss balancing methods can be incorporated into PhaVIP to overcome the imbalance problem. Second, although some variants of CGR incorporate polarity, charge, and molecular weight information for training (Dick and Green 2020), our results show that they have nearly the same results (performance difference <1.5%) as the original 20-flake CGRs. We will investigate whether there are other suitable features that our framework can utilize to improve performance. Third, we will explore whether we can employ the few-shot learning-based method to learn features from the classes with few labels. This can be used to provide more detailed annotations for further analyzing phages.

## Supplementary Material

btad229_Supplementary_Data

## Data Availability

All data and codes used for this study are available online via: https://phage.ee.cityu.edu.hk/phavip/download.

## References

[btad229-B1] Aksyuk AA , Rossmann MG. Bacteriophage assembly. Viruses 2011;3:172–203. 21994726 10.3390/v3030172 PMC3185693

[btad229-B2] Arif M , Ali F, Ahmad S et al Pred-BVP-Unb: fast prediction of bacteriophage virion proteins using un-biased multi-perspective properties with recursive feature elimination. Genomics 2020;112:1565–74. 31526842 10.1016/j.ygeno.2019.09.006

[btad229-B3] Azimi T , Mosadegh M, Nasiri MJ et al Phage therapy as a renewed therapeutic approach to mycobacterial infections: a comprehensive review. Infect Drug Resist 2019;12:2943–59. 31571947 10.2147/IDR.S218638 PMC6756577

[btad229-B4] Baevski A , Auli M. Adaptive input representations for neural language modeling. In: *International Conference on Learning Representations*. 2018.

[btad229-B5] Boeckaerts D , Stock M, Criel B et al Predicting bacteriophage hosts based on sequences of annotated receptor-binding proteins. Sci Rep 2021;11:1–14. 33446856 10.1038/s41598-021-81063-4 PMC7809048

[btad229-B6] Brüssow H , Desiere F. Comparative phage genomics and the evolution of siphoviridae: insights from dairy phages. Mol Microbiol 2001;39:213–22. 11136444 10.1046/j.1365-2958.2001.02228.x

[btad229-B7] Cantu VA , Salamon P, Seguritan V et al PhANNs, a fast and accurate tool and web server to classify phage structural proteins. PLoS Comput Biol 2020;16:e1007845. 33137102 10.1371/journal.pcbi.1007845 PMC7660903

[btad229-B8] Charoenkwan P , Kanthawong S, Schaduangrat N et al PVPred-SCM: improved prediction and analysis of phage virion proteins using a scoring card method. Cells2020a;9:353. 32028709 10.3390/cells9020353 PMC7072630

[btad229-B9] Charoenkwan P , Nantasenamat C, Hasan M et al Meta-iPVP: a sequence-based meta-predictor for improving the prediction of phage virion proteins using effective feature representation. J Comput Aided Mol Des2020b;34:1105–16. 32557165 10.1007/s10822-020-00323-z

[btad229-B10] Cobián Güemes AG , Youle M, Cantú VA et al Viruses as winners in the game of life. Annu Rev Virol 2016;3:197–214. 27741409 10.1146/annurev-virology-100114-054952

[btad229-B11] Devlin J , Chang M-W, Lee K et al Bert: pre-training of deep bidirectional transformers for language understanding. *arXiv preprint arXiv:1810.04805*. 2018.

[btad229-B12] Dick K , Green JR. Chaos game representations & deep learning for proteome-wide protein prediction. In: *2020 IEEE 20th International Conference on Bioinformatics and Bioengineering (BIBE)*, pp. 115–21. IEEE, 2020.

[btad229-B13] Ding H , Feng P-M, Chen W et al Identification of bacteriophage virion proteins by the ANOVA feature selection and analysis. Mol Biosyst 2014;10:2229–35. 24931825 10.1039/c4mb00316k

[btad229-B14] Dosovitskiy A , Beyer L, Kolesnikov A et al An image is worth 16×16 words: transformers for image recognition at scale. In: *International Conference on Learning Representations*. 2020.

[btad229-B15] Eloe-Fadrosh EA. Towards a genome-based virus taxonomy. Nat Microbiol 2019;4:1249–50. 31337890 10.1038/s41564-019-0511-9

[btad229-B16] Emerson JB , Thomas BC, Andrade K et al Dynamic viral populations in hypersaline systems as revealed by metagenomic assembly. Appl Environ Microbiol 2012;78:6309–20. 22773627 10.1128/AEM.01212-12 PMC3416638

[btad229-B17] Fang Z , Feng T, Zhou H et al DeePVP: identification and classification of phage virion proteins using deep learning. Gigascience 2022;11:giac076. 35950840 10.1093/gigascience/giac076 PMC9366990

[btad229-B18] Fang Z , Zhou H. VirionFinder: identification of complete and partial prokaryote virus virion protein from virome data using the sequence and biochemical properties of amino acids. Front Microbiol 2021;12:615711. 33613485 10.3389/fmicb.2021.615711 PMC7894196

[btad229-B19] Feng P-M , Ding H, Chen W et al Naive Bayes classifier with feature selection to identify phage virion proteins. Comput Math Methods Med 2013;2013:530696. 23762187 10.1155/2013/530696 PMC3671239

[btad229-B20] Fernández L , Rodríguez A, García P. Phage or foe: an insight into the impact of viral predation on microbial communities. ISME J 2018;12:1171–9. 29371652 10.1038/s41396-018-0049-5 PMC5932045

[btad229-B21] Fiser A , Tusnady GE, Simon I. Chaos game representation of protein structures. J Mol Graph 1994;12:302–4. 7696222 10.1016/0263-7855(94)80109-6

[btad229-B22] Ghiasi A , Kazemi H, Borgnia E et al What do vision transformers learn? A visual exploration. *arXiv preprint arXiv:2212.06727*. 2022.

[btad229-B23] Han H , Zhu W, Ding C et al iPVP-MCV: a multi-classifier voting model for the accurate identification of phage virion proteins. Symmetry 2021;13:1506.

[btad229-B24] Hoang T , Yin C, Yau SS-T. Numerical encoding of DNA sequences by chaos game representation with application in similarity comparison. Genomics 2016;108:134–42. 27538895 10.1016/j.ygeno.2016.08.002

[btad229-B25] Hockenberry AJ , Wilke CO. BACPHLIP: predicting bacteriophage lifestyle from conserved protein domains. PeerJ 2021;9:e11396. 33996289 10.7717/peerj.11396 PMC8106911

[btad229-B26] Howard-Varona C , Hargreaves KR, Abedon ST et al Lysogeny in nature: mechanisms, impact and ecology of temperate phages. ISME J 2017;11:1511–20. 28291233 10.1038/ismej.2017.16 PMC5520141

[btad229-B27] Hyatt D , Chen G-L, LoCascio PF et al Prodigal: prokaryotic gene recognition and translation initiation site identification. BMC Bioinformatics 2010;11:1–11. 20211023 10.1186/1471-2105-11-119 PMC2848648

[btad229-B28] Jeffrey HJ. Chaos game representation of gene structure. Nucleic Acids Res 1990;18:2163–70. 2336393 10.1093/nar/18.8.2163 PMC330698

[btad229-B29] Kabir M , Nantasenamat C, Kanthawong S et al Large-scale comparative review and assessment of computational methods for phage virion proteins identification. Excli J 2022;21:11–29. 35145365 10.17179/excli2021-4411 PMC8822302

[btad229-B30] Lee S-E , Lee D-Y, Lee W-G et al Osong public health and research perspectives. Osong Public Health Res Perspect 2019;10:295–306. 31673491 10.24171/j.phrp.2019.10.5.06 PMC6816357

[btad229-B31] Li W , Godzik A. Cd-hit: a fast program for clustering and comparing large sets of protein or nucleotide sequences. Bioinformatics 2006;22:1658–9. 16731699 10.1093/bioinformatics/btl158

[btad229-B32] Löchel HF , Eger D, Sperlea T et al Deep learning on chaos game representation for proteins. Bioinformatics 2020;36:272–9. 31225868 10.1093/bioinformatics/btz493

[btad229-B33] Löchel HF , Heider D. Chaos game representation and its applications in bioinformatics. Comput Struct Biotechnol J 2021;19:6263–71. 34900136 10.1016/j.csbj.2021.11.008 PMC8636998

[btad229-B34] Lyon J. Phage therapy’s role in combating antibiotic-resistant pathogens. JAMA 2017;318:1746–8. 29071339 10.1001/jama.2017.12938

[btad229-B35] Manavalan B , Shin TH, Lee G. PVP-SVM: sequence-based prediction of phage virion proteins using a support vector machine. Front Microbiol 2018;9:476. 29616000 10.3389/fmicb.2018.00476 PMC5864850

[btad229-B36] Meng C , Zhang J, Ye X et al Review and comparative analysis of machine learning-based phage virion protein identification methods. Biochim Biophys Acta Proteins Proteom 2020;1868:140406. 32135196 10.1016/j.bbapap.2020.140406

[btad229-B37] Menouni R , Hutinet G, Petit M-A et al Bacterial genome remodeling through bacteriophage recombination. FEMS Microbiol Lett 2015;362:1–10. 10.1093/femsle/fnu022 25790500

[btad229-B38] Nami Y , Imeni N, Panahi B. Application of machine learning in bacteriophage research. BMC Microbiol 2021;21:1–8. 34174831 10.1186/s12866-021-02256-5 PMC8235560

[btad229-B39] Pan Y , Gao H, Lin H et al Identification of bacteriophage virion proteins using multinomial naive Bayes with g-gap feature tree. IJMS 2018;19:1779. 29914091 10.3390/ijms19061779 PMC6032154

[btad229-B40] Petti S , Eddy SR. Constructing benchmark test sets for biological sequence analysis using independent set algorithms. PLoS Comput Biol 2022;18:e1009492. 35255082 10.1371/journal.pcbi.1009492 PMC8929697

[btad229-B41] Raghu M , Unterthiner T, Kornblith S et al Do vision transformers see like convolutional neural networks? Adv Neural Inf Process Syst 2021;34:12116–28.

[btad229-B42] Ren Y , Chakraborty T, Doijad S et al Prediction of antimicrobial resistance based on whole-genome sequencing and machine learning. Bioinformatics 2022;38:325–34. 34613360 10.1093/bioinformatics/btab681 PMC8722762

[btad229-B43] Roux S , Enault F, Hurwitz BL et al VirSorter: mining viral signal from microbial genomic data. PeerJ 2015;3:e985. 26038737 10.7717/peerj.985 PMC4451026

[btad229-B44] Ru X , Li L, Wang C. Identification of phage viral proteins with hybrid sequence features. Front Microbiol 2019;10:507. 30972038 10.3389/fmicb.2019.00507 PMC6443926

[btad229-B45] Seguritan V , Alves N Jr, Arnoult M et al Artificial neural networks trained to detect viral and phage structural proteins. PLoS Comput Biol 2012;8(8):e1002657. 10.1371/journal.pcbi.1002657 PMC3426561 22927809

[btad229-B46] Shang J , Sun Y. Predicting the hosts of prokaryotic viruses using GCN-based semi-supervised learning. BMC Biol 2021;19:1–15. 34819064 10.1186/s12915-021-01180-4 PMC8611875

[btad229-B47] Shang J , Sun Y. CHERRY: a computational metHod for accuratE pRediction of virus–pRokarYotic interactions using a graph encoder–decoder model. Brief Bioinform 2022;23:bbac182. 35595715 10.1093/bib/bbac182 PMC9487644

[btad229-B48] Sinha A , Eniyan K, Manohar P et al Characterization and genome analysis of B1 sub-cluster mycobacteriophage PDRPxv. Virus Res 2020;279:197884. 31981773 10.1016/j.virusres.2020.197884

[btad229-B49] Steinbacher S , Baxa U, Miller S et al Crystal structure of phage P22 tailspike protein complexed with Salmonella sp. O-antigen receptors. Proc Natl Acad Sci USA 1996;93:10584–8. 8855221 10.1073/pnas.93.20.10584 PMC38196

[btad229-B50] Stummeyer K , Dickmanns A, Mühlenhoff M et al Crystal structure of the polysialic acid–degrading endosialidase of bacteriophage K1F. Nat Struct Mol Biol 2005;12:90–6. 15608653 10.1038/nsmb874

[btad229-B51] Tan J-X , Dao F-Y, Lv H et al Identifying phage virion proteins by using two-step feature selection methods. Molecules 2018;23:2000. 30103458 10.3390/molecules23082000 PMC6222849

[btad229-B52] Vaswani A , Shazeer N, Parmar N et al Attention is all you need. In: *Advances in Neural Information Processing Systems*, pp. 5998–6008. 2017.

[btad229-B53] Wang L-F , Yu M. Epitope identification and discovery using phage display libraries: applications in vaccine development and diagnostics. Curr Drug Targets 2004;5:1–15. 14738215 10.2174/1389450043490668

[btad229-B54] Wang Q , Li B, Xiao T et al Learning deep transformer models for machine translation. In: *Proceedings of the 57th Annual Meeting of the Association for Computational Linguistics*, pp. 1810–22. 2019.

[btad229-B55] Zhang L , Zhang C, Gao R et al An ensemble method to distinguish bacteriophage virion from non-virion proteins based on protein sequence characteristics. Int J Mol Sci 2015;16:21734–58. 26370987 10.3390/ijms160921734 PMC4613277

[btad229-B56] Zhu Y , Shang J, Peng C et al Phage family classification under Caudoviricetes: a review of current tools using the latest ICTV classification framework. Front Microbiol 2022;13:1032186. 36590402 10.3389/fmicb.2022.1032186 PMC9800612

