# Supplementary material for: PhaVIP: Phage VIrion Protein classification based on chaos game representation and Vision Transformer
Source: Bioinformatics. 2023 Jun 30;39(Suppl 1):i30–9. doi: 10.1093/bioinformatics/btad229 (PMC10311294; doi:10.1093/bioinformatics/btad229)
Supplement: btad229_Supplementary_Data [file btad229_supplementary_data.pdf]

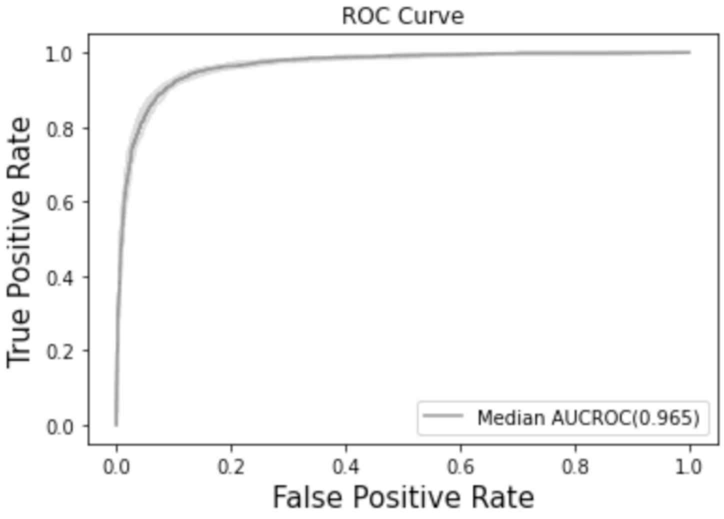

Figure S1. The ROC curve of the ten-fold cross-validation on the training set with the median as the solid line and the percentiles as the shadows.

Table S1. Binary classification - dataset split by time

	Tools	Precision	Recall	F1-score	No. of train	No. of test
PVP	PhANNs	0.81	0.87	0.84	27704	7509
	VirionFinder	0.77	0.92	0.84		
	DeePVP	0.99	0.51	0.68		
	Meta-iPVP	0.76	0.84	0.8		
	PVP-SVM	0.89	0.44	0.59		
	PVPred_SCM	0.78	0.57	0.66		
	PhaVIP	0.93	0.95	0.94		
Non-PVP	PhANNs	0.86	0.8	0.83	27704	7509
	VirionFinder	0.9	0.73	0.81		
	DeePVP	0.67	1	0.8		
	Meta-iPVP	0.82	0.73	0.77		
	PVP-SVM	0.63	0.94	0.75		
	PVPred_SCM	0.66	0.84	0.74		
	PhaVIP	0.95	0.93	0.94		

Table S2 multi-class classification - dataset split by time

	Tools	Precision	Recall	F1-score	No. of train	No. of test
Major capsid	PhANNs	0.78	0.76	0.77	1942	507
	DeePVP	0.84	0.69	0.76		
	PhaVIP	0.89	0.85	0.87		
Minor capsid	PhANNs	0.14	0.58	0.23	360	38
	DeePVP	0.32	0.61	0.41		
	PhaVIP	0.62	0.53	0.57		
Baseplate	PhANNs	0.86	0.82	0.84	2646	978
	DeePVP	0.99	0.72	0.84		
	PhaVIP	0.96	0.93	0.94		
Major tail	PhANNs	0.56	0.7	0.63	1123	335
	DeePVP	0.94	0.58	0.72		
	PhaVIP	0.9	0.85	0.87		
Minor tail	PhANNs	0.88	0.66	0.75	360	38
	DeePVP	0.87	0.82	0.84		
	PhaVIP	0.92	0.95	0.93		
Protal	PhANNs	0.71	0.82	0.76	2206	564
	DeePVP	0.83	0.78	0.81		
	PhaVIP	0.93	0.96	0.94		
Tail fiber	PhANNs	0.6	0.75	0.67	1912	521
	DeePVP	0.64	0.84	0.73		
	PhaVIP	0.84	0.88	0.86		

Table S3 binary classification - dataset split by similarity (threshold=0.4)

	Tools	Precision	Recall	F1-score	No. of train	No. of test
PVP	PhANNs	0.71	0.93	0.8	8671	7869
	DeePVP	0.88	0.76	0.82		
	PhaVIP	0.89	0.85	0.85		
Non-PVP	PhANNs	0.89	0.62	0.73	8671	7869
	DeePVP	0.84	0.97	0.9		
	PhaVIP	0.87	0.92	0.89		

Table S4 binary classification - dataset split by similarity (threshold=0.5)

	Tools	Precision	Recall	F1-score	No. of train	No. of test
PVP	PhANNs	0.79	0.81	0.8	9112	8361
	DeePVP	0.94	0.7	0.81		
	PhaVIP	0.85	0.84	0.85		
Non-PVP	PhANNs	0.8	0.79	0.8	9112	8361
	DeePVP	0.76	0.96	0.85		
	PhaVIP	0.84	0.86	0.85		

Table S5 binary classification - dataset split by similarity (threshold=0.6)

	Tools	Precision	Recall	F1-score	No. of train	No. of test
PVP	PhANNs	0.81	0.85	0.83	9372	8251
	DeePVP	0.75	0.92	0.83		
	PhaVIP	0.9	0.78	0.84		
Non-PVP	PhANNs	0.84	0.79	0.82	9372	8251
	DeePVP	0.94	0.69	0.79		
	PhaVIP	0.84	0.93	0.88		

Table S6 binary classification - dataset split by similarity (threshold=0.7)

	Tools	Precision	Recall	F1-score	No. of train	No. of test
PVP	PhANNs	0.82	0.87	0.84	9239	8521
	DeePVP	0.91	0.8	0.85		
	PhaVIP	0.9	0.83	0.87		
Non-PVP	PhANNs	0.86	0.8	0.83	9239	8521
	DeePVP	0.82	0.96	0.88		
	PhaVIP	0.87	0.92	0.89		

Table S7 binary classification - dataset split by similarity (threshold=0.8)

	Tools	Precision	Recall	F1-score	No. of train	No. of test
PVP	PhANNs	0.86	0.85	0.85	9197	8662
	DeePVP	0.92	0.84	0.87		
	PhaVIP	0.92	0.85	0.88		
Non-PVP	PhANNs	0.85	0.86	0.85	9197	8662
	DeePVP	0.86	0.94	0.9		
	PhaVIP	0.88	0.93	0.91		

Table S8 binary classification - dataset split by similarity (threshold=0.9)

	Tools	Precision	Recall	F1-score	No. of train	No. of test
PVP	PhANNs	0.86	0.86	0.86	9263	8668
	DeePVP	0.88	0.9	0.89		
	PhaVIP	0.92	0.86	0.89		
Non-PVP	PhANNs	0.86	0.86	0.86	9263	8668
	DeePVP	0.91	0.88	0.9		
	PhaVIP	0.89	0.94	0.91		

Table S9 multi-class classification - dataset split by similarity (threshold=0.4)

	Tools	Precision	Recall	F1-score	No. of train	No. of test
Major capsid	PhANNs	0.48	0.56	0.52	515	1137
	DeePVP	0.53	0.25	0.34		
	PhaVIP	0.58	0.52	0.55		
Minor capsid	PhANNs	0.16	0.45	0.23	189	184
	DeePVP	0.33	0.12	0.18		
	PhaVIP	0.42	0.39	0.4		
Baseplate	PhANNs	0.66	0.53	0.58	1981	1401
	DeePVP	0.56	0.26	0.35		
	PhaVIP	0.57	0.61	0.59		
Major tail	PhANNs	0.3	0.35	0.32	797	633
	DeePVP	0.59	0.32	0.41		
	PhaVIP	0.58	0.52	0.55		
Minor tail	PhANNs	0.75	0.52	0.61	2430	2399
	DeePVP	0.74	0.49	0.59		
	PhaVIP	0.82	0.69	0.75		
Protal	PhANNs	0.47	0.56	0.51	1469	1193
	DeePVP	0.42	0.73	0.53		
	PhaVIP	0.66	0.8	0.72		
	PhANNs	0.41	0.49	0.44		

Tail fiber	DeePVP	0.19	0.73	0.31	1290	922
	PhaVIP	0.57	0.37	0.44		

Table S10 multi-class classification - dataset split by similarity (threshold=0.5)

	Tools	Precision	Recall	F1-score	No. of train	No. of test
	PhANNs	0.52	0.57	0.54		
Major capsid	DeePVP	0.56	0.57	0.57	1198	1195
	PhaVIP	0.64	0.5	0.56		
	PhANNs	0.22	0.6	0.32		
Minor capsid	DeePVP	0.23	0.1	0.14	191	186
	PhaVIP	0.21	0.16	0.18		
	PhANNs	0.57	0.38	0.46		
Baseplate	DeePVP	0.69	0.21	0.32	1912	1470
	PhaVIP	0.6	0.55	0.57		
	PhANNs	0.32	0.37	0.34		
Major tail	DeePVP	0.36	0.25	0.3	762	665
	PhaVIP	0.64	0.5	0.56		
	PhANNs	0.71	0.55	0.62		
Minor tail	DeePVP	0.59	0.75	0.66	2499	2408
	PhaVIP	0.79	0.76	0.78		
	PhANNs	0.56	0.76	0.65		
Protal	DeePVP	0.43	0.78	0.56	1393	1284
	PhaVIP	0.67	0.88	0.76		
	PhANNs	0.52	0.52	0.52		
Tail fiber	DeePVP	0.38	0.66	0.48	1157	1153
	PhaVIP	0.56	0.5	0.53		

Table S11 multi-class classification - dataset split by similarity (threshold=0.6)

	Tools	Precision	Recall	F1-score	No. of train	No. of test
	PhANNs	0.56	0.57	0.57		
Major capsid	DeePVP	0.47	0.33	0.38	1241	1145
	PhaVIP	0.62	0.42	0.5		
	PhANNs	0.19	0.52	0.28		
Minor capsid	DeePVP	0.5	0.12	0.19	194	185
	PhaVIP	0.22	0.26	0.24		
	PhANNs	0.71	0.46	0.56		
Baseplate	DeePVP	0.88	0.63	0.73	1840	1613
	PhaVIP	0.77	0.7	0.74		
	PhANNs	0.39	0.51	0.44		
Major tail	DeePVP	0.3	0.34	0.32	752	694

	PhaVIP	0.62	0.42	0.5		
	PhANNs	0.79	0.53	0.64		
Minor tail	DeePVP	0.69	0.61	0.65	2576	2343
	PhaVIP	0.78	0.77	0.78		
	PhANNs	0.56	0.7	0.62		
Protal	DeePVP	0.5	0.8	0.62	1509	1172
	PhaVIP	0.66	0.83	0.74		
	PhANNs	0.4	0.58	0.47		
Tail fiber	DeePVP	0.38	0.8	0.52	1260	1099
	PhaVIP	0.58	0.65	0.62		

Table S12 multi-class classification - dataset split by similarity (threshold=0.7)

	Tools	Precision	Recall	F1-score	No. of train	No. of test
	PhANNs	0.6	0.62	0.61		
Major capsid	DeePVP	0.47	0.33	0.38	1265	1134
	PhaVIP	0.67	0.8	0.73		
	PhANNs	0.19	0.67	0.29		
Minor capsid	DeePVP	0.5	0.12	0.19	190	189
	PhaVIP	0.48	0.15	0.23		
	PhANNs	0.72	0.5	0.59		
Baseplate	DeePVP	0.88	0.63	0.73	1933	1595
	PhaVIP	0.75	0.69	0.72		
	PhANNs	0.39	0.44	0.42		
Major tail	DeePVP	0.3	0.73	0.43	718	714
	PhaVIP	0.67	0.8	0.73		
	PhANNs	0.75	0.62	0.68		
Minor tail	DeePVP	0.69	0.61	0.65	2510	2440
	PhaVIP	0.81	0.75	0.77		
	PhANNs	0.62	0.7	0.66		
Protal	DeePVP	0.5	0.8	0.62	1353	1337
	PhaVIP	0.76	0.87	0.81		
	PhANNs	0.49	0.55	0.52		
Tail fiber	DeePVP	0.38	0.8	0.52	1270	1112
	PhaVIP	0.63	0.62	0.63		

Table S13 multi-class classification - dataset split by similarity (threshold=0.8)

	Tools	Precision	Recall	F1-score	No. of train	No. of test
	PhANNs	0.58	0.63	0.61		
Major capsid	DeePVP	0.5	0.25	0.33	1242	1163
	PhaVIP	0.73	0.46	0.75		
	PhANNs	0.21	0.63	0.31		
Minor capsid	DeePVP	0.25	0.51	0.36	191	188

	PhaVIP	0.47	0.37	0.41		
	PhANNs	0.79	0.52	0.63		
Baseplate	DeePVP	0.75	0.62	0.68	1814	1736
	PhaVIP	0.73	0.71	0.72		
	PhANNs	0.38	0.4	0.39		
Major tail	DeePVP	0.54	0.29	0.38	773	667
	PhaVIP	0.73	0.46	0.75		
	PhANNs	0.75	0.67	0.71		
Minor tail	DeePVP	0.55	0.9	0.68	2513	2469
	PhaVIP	0.81	0.82	0.81		
	PhANNs	0.66	0.71	0.68		
Protal	DeePVP	0.67	0.69	0.68	1385	1323
	PhaVIP	0.78	0.83	0.81		
	PhANNs	0.44	0.53	0.48		
Tail fiber	DeePVP	0.54	0.57	0.56	1279	1116
	PhaVIP	0.59	0.65	0.62		

Table S14 multi-class classification - dataset split by similarity (threshold=0.9)

	Tools	Precision	Recall	F1-score	No. of train	No. of test
	PhANNs	0.59	0.62	0.6		
Major capsid	DeePVP	0.65	0.4	0.5	1279	1129
	PhaVIP	0.79	0.76	0.77		
	PhANNs	0.28	0.53	0.36		
Minor capsid	DeePVP	0.31	0.42	0.36	192	187
	PhaVIP	0.41	0.32	0.36		
	PhANNs	0.74	0.57	0.64		
Baseplate	DeePVP	0.87	0.7	0.78	1851	1704
	PhaVIP	0.78	0.81	0.79		
	PhANNs	0.45	0.54	0.49		
Major tail	DeePVP	0.81	0.16	0.27	739	695
	PhaVIP	0.79	0.65	0.72		
	PhANNs	0.86	0.61	0.71		
Minor tail	DeePVP	0.78	0.86	0.82	2529	2502
	PhaVIP	0.86	0.86	0.86		
	PhANNs	0.59	0.8	0.68		
Protal	DeePVP	0.58	0.94	0.72	1403	1315
	PhaVIP	0.84	0.94	0.88		
	PhANNs	0.47	0.6	0.53		
Tail fiber	DeePVP	0.56	0.76	0.65	1270	1136
	PhaVIP	0.69	0.69	0.69		
